# Comparative transcriptomics analysis of tolerant and sensitive genotypes reveals genes involved in the response to cold stress in bitter gourd (*Momordica charantia* L.)

**DOI:** 10.1038/s41598-024-58754-9

**Published:** 2024-07-17

**Authors:** Yu Ning, Zhiyang Liu, Jing Liu, Renjie Qi, Pengfei Xia, Xihan Yuan, Hai Xu, Longzheng Chen

**Affiliations:** 1 Jiangsu Key Laboratory for Horticultural Crop Genetic Improvement, Institute of Vegetable Crops, Jiangsu Academy of Agricultural Sciences, Nanjing, 210014 China; 2 College of Pharmacy, Jiangsu Ocean University, Lianyungang, 222005 China; 3 Nanjing Innovation Vegetable Molecular Breeding Research Institute, Nanjing, 211899 China

**Keywords:** Plant hormones, Plant stress responses

## Abstract

Bitter gourd is an economically important horticultural crop for its edible and medicinal value. However, the regulatory mechanisms of bitter gourd in response to cold stress are still poorly elucidated. In this study, phytohormone determination and comparative transcriptome analyses in XY (cold-tolerant) and QF (cold-sensitive) after low temperature treatment were conducted. Under cold stress, the endogenous contents of abscisic acid (ABA), jasmonic acid (JA) and salicylic acid (SA) in XY were significantly increased at 24 h after treatment (HAT), indicating that ABA, JA and SA might function in regulating cold resistance. RNA-seq results revealed that more differentially expressed genes were identified at 6 HAT in QF and 24 HAT in XY, respectively. KEGG analysis suggested that the plant hormone signal transduction pathway was significantly enriched in both genotypes at all the time points. In addition, transcription factors showing different expression patterns between XY and QF were identified, including *CBF3*, *ERF2*, *NAC90*, *WRKY51* and *WRKY70*. Weighted gene co-expression network analysis suggested *MARK1*, *ERF17*, *UGT74E2*, *GH3.1* and *PPR* as hub genes. These results will deepen the understanding of molecular mechanism of bitter gourd in response to cold stress and the identified genes may help to facilitate the genetic improvement of cold-resistant cultivars.

## Introduction

Low temperature, one of the major abiotic stresses in agricultural production, can negatively affect plant growth and development, decrease crop yield and limit geographical distribution and growing season^1^. Most species growing in the tropics and subtropical regions are sensitive to cold stress^2^. Plants will present various symptoms when exposed to low temperature, including repressed or delayed germination, stunted buds, wilting/yellowing of leaves, and even tissue death^3^. Cold stress can also induce a series of physiological and biochemical reactions in vivo, such as decreased membrane fluidity and stability, inhibited photosynthesis and excessive accumulation of reactive oxygen species (ROS)^4,5^.

Plants have evolved a series of sophisticated mechanisms to defend against cold stress and relieve cold injury to some extent^6^. Antioxidant enzymes such as catalase (CAT), superoxide dismutase (SOD), ascorbate peroxidase (APX) and peroxidase (POD) are activated to scavenge accumulated ROS and maintain redox balance^7^. Some osmoprotectants, including soluble sugar, glycinebetaine and proline, also accumulate to stabilize membrane lipids, maintain homeostasis and reduce electrolyte leakage and oxidative stress^8^. In addition, plant hormones also play important roles in mediating plant defense responses against cold stress. Studies have shown that low temperature can induce the expression of abscisic acid (ABA) biosynthesis genes and enhance plant cold tolerance by elevating the endogenous ABA content^9^. Cold stress can also lead to the accumulation of endogenous salicylic acid (SA), and exogenous SA treatment significantly enhances the cold tolerance of maize^10^. Moreover, jasmonic acid (JA) participates in responses to cold stress by acting as a positive regulator^11^.

In recent years, the molecular regulation mechanisms underlying cold tolerance have been comprehensively investigated, and large numbers of genes responding to low temperature have been identified. The ICE-CBF-COR transcriptional regulatory cascade is one of the most thoroughly studied pathways responsive to cold stress. C-repeat binding factors (CBFs)/Dehydration-responsive element-binding proteins (DREBs), members of the AP2/ERF family, can` be activated by inducer of CBF expression protein (ICE)^12^. Then, *CBFs* induce the expression of cold-responsive (*COR*) genes by binding to *cis*-elements in the promoter of *CORs*^13^. Plants also evolve CBF-independent pathways to adapt to cold stress, such as *HOS10* (an R2R3-type MYB), which probably regulates ABA-dependent cold acclimation pathways by positively regulating *NCED3*^14^. Although large numbers of cold-responsive genes have been identified in several plant species, the complete cold-responsive mechanism has not been elucidated.

With the rapid development of next-generation sequencing (NGS) technologies, high-throughput RNA-seq makes it possible to obtain a general overview of transcript profiles and gene dynamic changes in both model and non-model species. To date, RNA-seq has been widely applied to identify cold-responsive genes and analyze molecular mechanism of cold stress tolerance in a mass of crops, such as rice^15^, maize^16^, cotton^17^, rapeseed^18^, bell pepper^19^, apple^20^ and castor^21^. In Cucurbitaceae family, RNA-seq also play important roles in mining genes in response to cold stress. Comparative transcriptome analysis between resistant and sensitive cucumber lines suggested lower transcription levels of genes involved in plant hormone transductions and strongly upregulated *cinnamyl-alcohol dehydrogenase* (*CAD*) genes in the resistant genotype^22^. In pumpkin, genes in the plant hormone signal transduction pathway and transcription factor including *AP2/ERF*, *bHLH*, *WRKY*, *MYB* and *HSF* were activated after low temperature treatment^23^.

Bitter gourd (*Momordica charantia* L., 2n = 2 ×  = 22) is an important kind of Cucurbitaceae crops and widely cultivated within tropical and subtropical regions^24^. Several studies have revealed the medicinal effects of bitter gourd, including antidiabetic, antiviral, antitumor, antibacterial, antioxidant, antiulcer, anti-inflammatory, hypotensive and immunostimulant properties^25,26^. With the expansion of the cultivation toward northern areas, low temperature becomes a pivotal factor affecting bitter gourd industry. Therefore, it is of great importance to obtain a better understanding of the molecular mechanism of cold tolerance and identify key genes in response to low temperature in bitter gourd to establish a theoretical basis to facilitate further resistance breeding. However, genes associated with cold tolerance and molecular mechanisms in response to cold stress in bitter gourd have been poorly elucidated. Comparative transcriptome analysis between Y17 (cold-susceptible) and Y54 (cold-resistant) revealed higher expression levels of genes encoding antioxidant enzymes and some transcription factors in cold resistant genotype^27^. Therefore, continuous identification of cold-responsive regulatory pathways and genes is still necessary and indispensable.

In the present study, comparative physiological and RNA-seq analyses between cold-sensitive and cold-tolerant bitter gourd genotypes were performed at 6 and 24 h after cold treatment, aiming at to clarify two major issues: i) which pathways and genes are involved in the response to cold stress at different time points and ii) which genes may cause cold resistance differences at the transcriptional level between different resistant genotypes. Our findings provide foundation for further elucidation of the molecular mechanisms responsible for cold stress resistance in bitter gourd.

## Results

### Morphological and phytohormone changes of bitter gourd under cold stress

**Figure 1:**
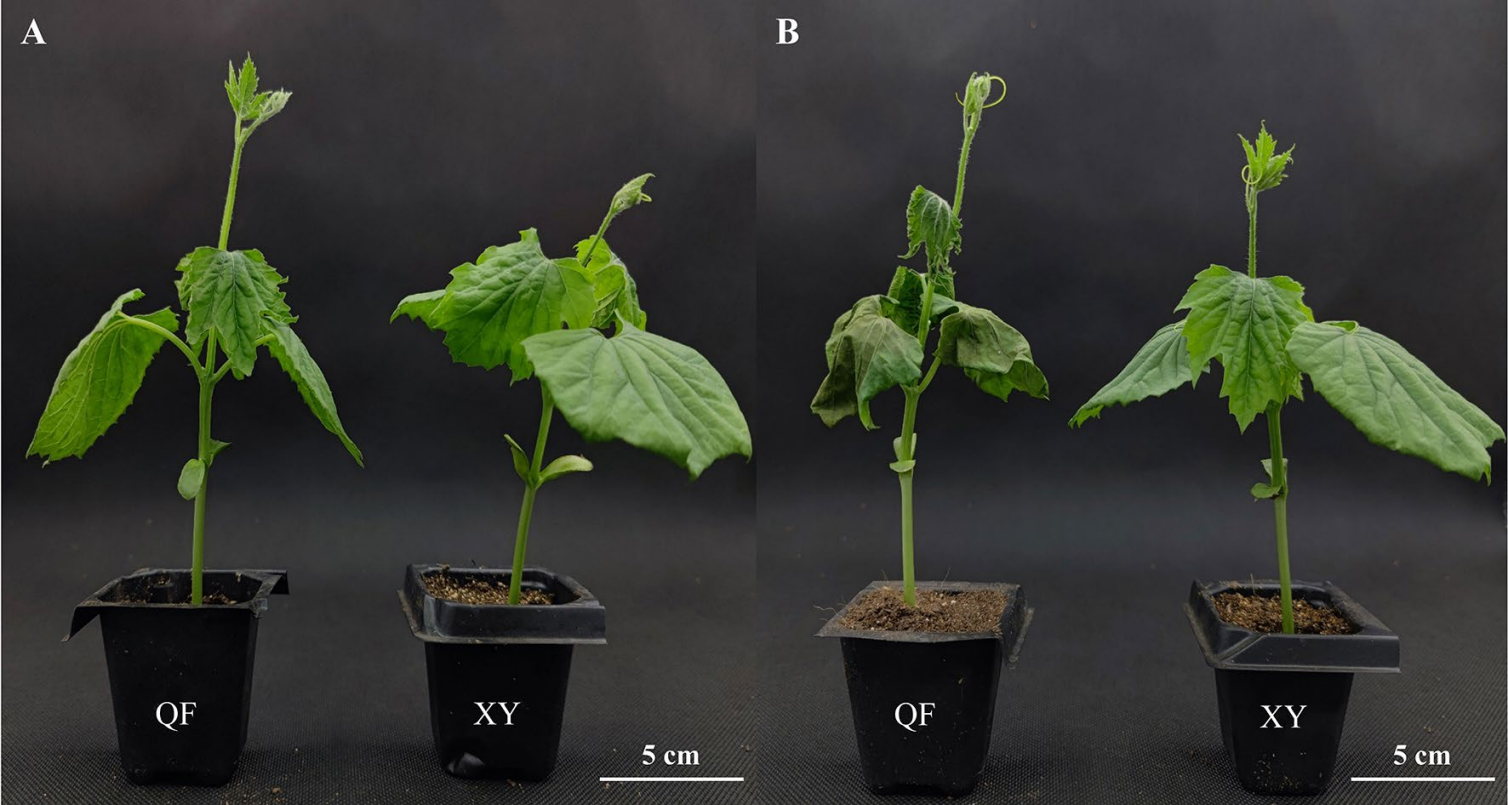
Phenotypic responses of the cold-sensitive (QF) and cold-tolerant (XY) genotypes after cold treatment at 6 h (**A**) and 24 h (**B**).

First, the phenotypes of XY and QF in response to cold stress were observed and compared. At 6 HAT, the young leaves of QF became slightly curved, while no obvious change could be observed in XY (Fig. 1A). At 24 HAT, severe injury symptoms could be observed in QF and the leaves became wilted and chlorosis. However, XY still showed no apparent damage under cold stress (Fig. 1B). Therefore, it could be concluded that XY was much more tolerant than QF against low temperature and these two genotypes could be used as two contrasting materials for further RNA-seq.

**Figure 2:**
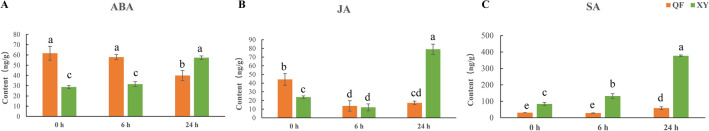
Changes of endogenous hormone levels in QF and XY under cold stress. (**A**) Abscisic acid. (**B**) Jasmonic acid. (**C**) Salicylic acid. Columns marked with different lowercase letters mean significant differences (*p* ≤ 0.05) among time points or genotypes.

Moreover, to elucidate phytohormone changes in bitter gourd in response to cold stress, endogenous ABA, JA and SA contents of both XY and QF were analyzed by HPLC/MS. Compared with 0 h, the endogenous ABA content of XY was significantly elevated at 24 HAT; however, it was significantly decreased at the same time point in QF (Fig. 2A). The endogenous JA contents of QF at both 6 and 24 HAT were much lower than that at 0 h; however, the JA level of XY at 24 HAT was significantly higher than that at 0 h (Fig. 2B). After cold treatment, the endogenous SA content increased in both materials at 6 and 24 HAT, and the SA content of XY was significantly higher than that of QF at each time point (Fig. 2C). In conclusion, cold stress triggered different hormonal responses between XY and QF.

### Transcriptome sequencing and data analysis

To reveal the molecular mechanism of the cold tolerance difference between XY and QF at the early stress stages, gene expression profiles of both genotypes at 0, 6 and 24 h after cold treatment were analyzed by RNA-seq with three biological replicates. 18 cDNA libraries were constructed and sequenced using the Illumina NovaSeq platform. Approximately, 43 ~ 54 million raw reads were generated from each library (Supplementary Table S1). All raw reads were deposited in the NCBI database with an SRA accession number PRJNA943061. After removing ambiguous nucleotides, low quality sequences and contaminated sequences, each library could generate 44.62 million clean reads, which were greater than 6.69 Gb on average. For each library, the GC content was ~ 47%, and the Q30 percentage was greater than 92.59% (Supplementary Table S1). Approximately 92.64% to 97.87% clean reads in each library could be mapped to the bitter gourd reference genome, of which 82.85% to 88.81% were uniquely mapped.

### Identification of differentially expressed genes (DEGs) induced by cold stress

**Figure 3:**
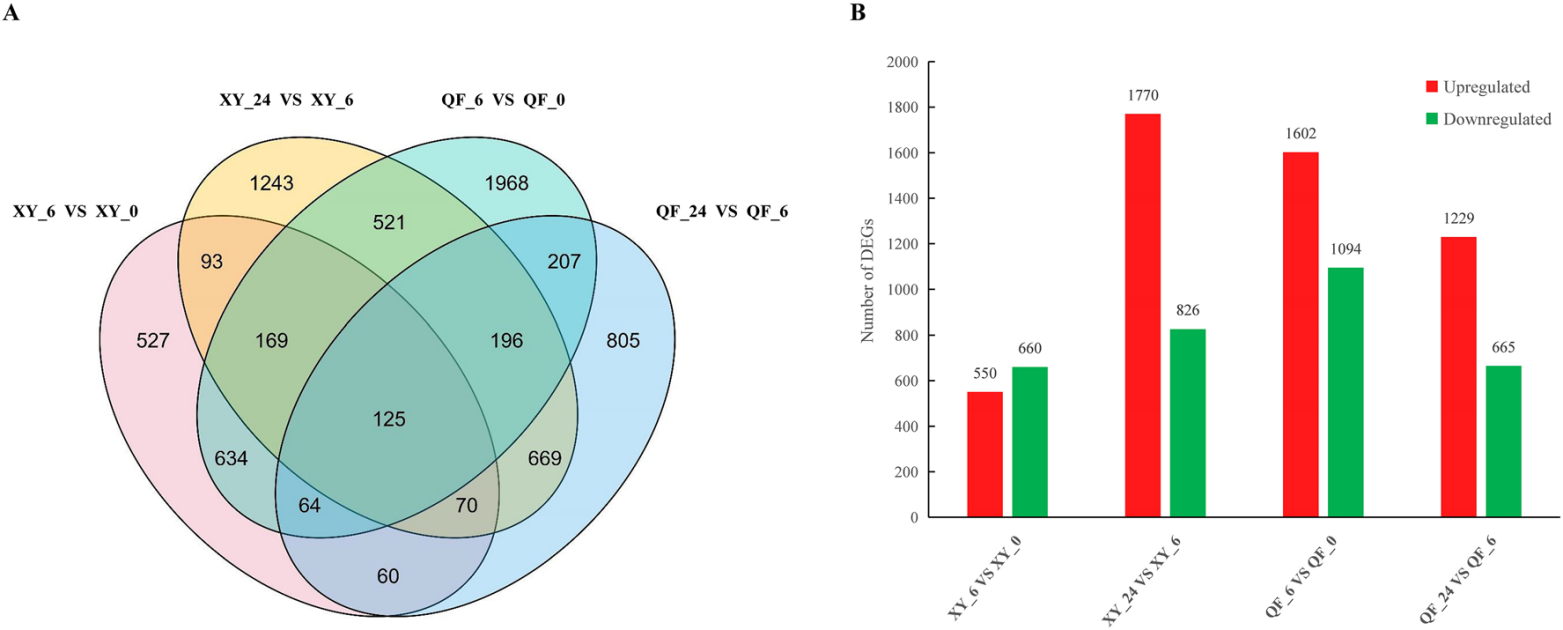
Overview of the identified DEGs by pairwise comparisons of transcriptomes of 0 HAT vs 6 HAT, or 6 HAT vs 24 HAT for each genotype. (**A**) Venn diagram. (**B**) Histograms of number of upregulated or downregulated DEGs.

To investigate the changes of transcripts abundance in response to cold stress, differentially expressed genes were identified and analyzed by comparing libraries at 6 HAT to those at 0 HAT, or comparing libraries at 24 HAT to those at 6 HAT. Without considering time points and genotypes, a total of 7,351 genes were differentially expressed under cold stress (Fig. 3A). For XY, 1,210 DEGs (550 upregulated and 660 downregulated) were identified at 6 HAT; however, 2,596 genes (1,770 upregulated and 826 downregulated) were differentially expressed at 24 HAT. For QF, 2,696 DEGs (1,602 upregulated and 1,094 downregulated) were identified at 6 HAT, and 1,894 genes (1,229 upregulated and 665 downregulated) were differentially expressed at 24 HAT (Fig. 3B). In conclusion, greater numbers of DEGs were identified in the cold-sensitive QF at 6 HAT, in contrast, more genes were differentially expressed in the cold-tolerant XY at 24 HAT.

### Functional annotation and enrichment analysis of DEGs associated with cold stress

**Figure 4:**
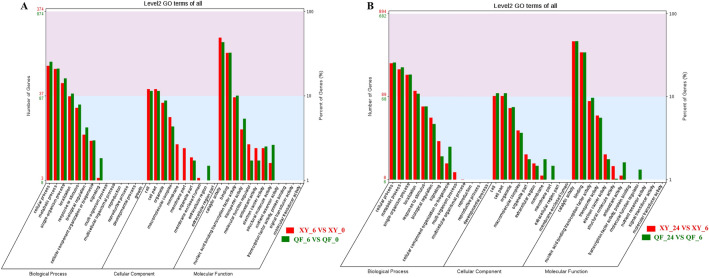
GO enrichment analysis of DEGs in response to cold treatment (4 °C) at 6 HAT (**A**) and 24 HAT (**B**). The left and right y-axis represent the number of DEGs and the percentage of DEGs in each category, respectively. The x-axis show the names of GO terms.

To obtain insight into the functional significance of the DEGs between XY and QF under low temperature, all the identified DEGs were subjected to the GO database to classify into three catagories: biological process (BP), cellular component (CC) and molecular function (MF). The results suggested that “cellular process”, “metabolic process”, “single-organism process”, “localization”, “cell”, “cell part”, “organelle”, “catalytic activity”, “binding” and “nucleic acid binding transcription factor activity” were the top ten significantly enriched GO terms shared in both genotypes and time points (Fig. 4). Interestingly, “membrane part” was exclusively enriched in XY at 6 HAT (9 DEGs) but was solely enriched in QF at 24 HAT (10 DEGs), showing an entirely different enrichment pattern. Moreover, “membrane” (10 DEGs) and “extracellular region” (13 DEGs) were especially enriched in XY and QF at 6 HAT, respectively.

**Figure 5:**
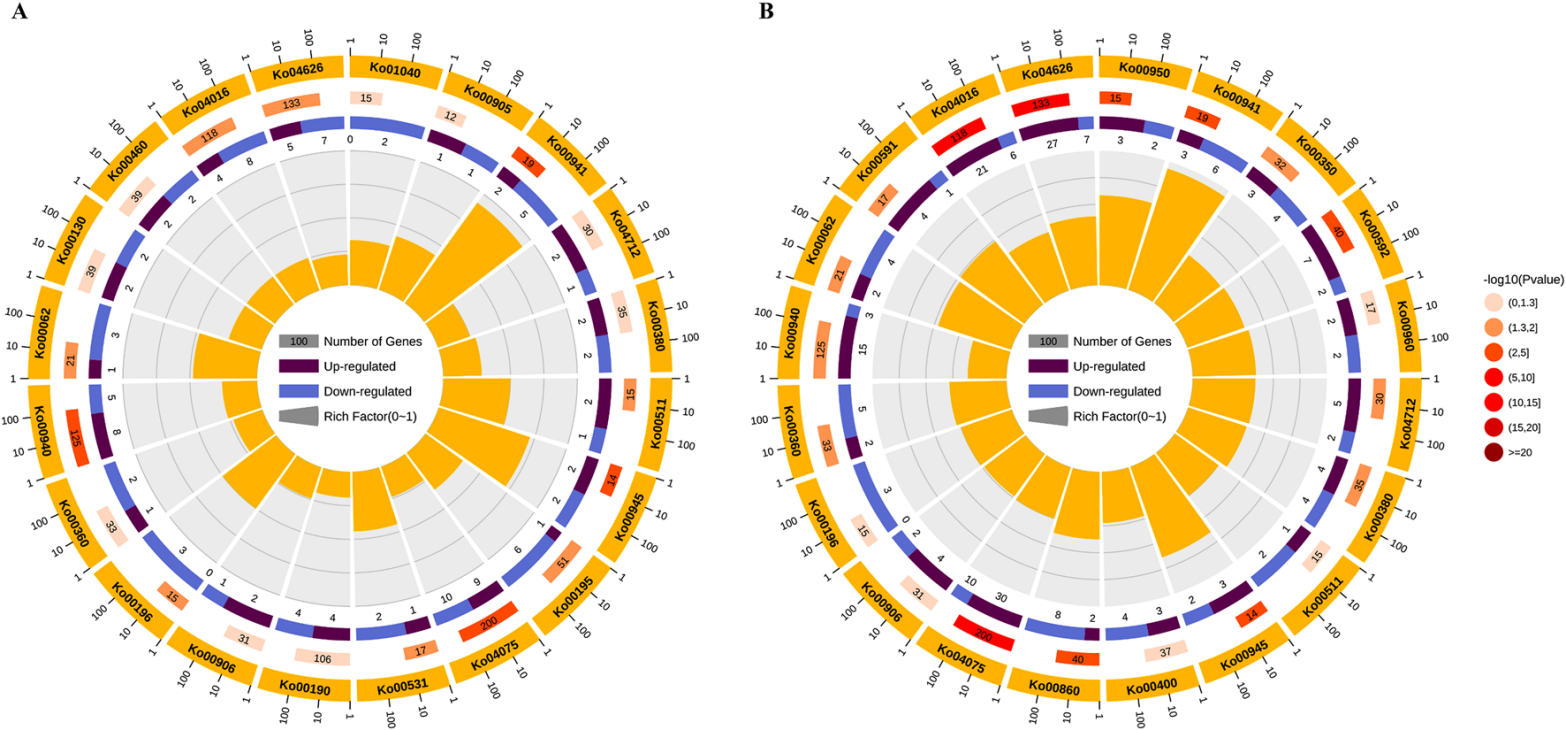
KEGG pathway analyses of DEGs in XY (**A**) and QF (**B**) under cold stress at 6 HAT. Circles from outer to inner sides indicate: 1. Entry numbers of enriched pathways; 2. Number of all genes within particular annotated pathway; 3. Number of DEGs within particular annotated pathway, the purple block mean the upregulated genes, the blue block represent the downregulated genes; 4. Rich factor of particular pathway.

In addition, to further understand the metabolic or signal pathways involved in responses to cold stress, all DEGs were mapped to the Kyoto Encyclopedia of Genes and Genomes (KEGG) database to retrieve the involved pathways, and the top 20 enriched pathways of XY and QF at 6 HAT (Fig. 5) and 24 HAT (Supplementary Fig. S1) were presented. A total of 30 pathways were significantly enriched (*p* value < 0.05) in the present study without considering genotypes or time points (Supplementary Table S2). At 6 HAT, “Flavonoid biosynthesis (Ko00941)”, “Plant hormone signal transduction (Ko04075)”, “Stilbenoid, diarylheptanoid and gingerol biosynthesis (Ko00945)”, “Phenylpropanoid biosynthesis (Ko00940)”, “MAPK signaling pathway-plant (Ko04016)”, “Fatty acid elongation(Ko00062)” and “Plant-pathogen interaction (Ko04626)” were significantly enriched in both XY and QF. However, DEGs showed different expression patterns between genotypes in common enriched pathways. For example, most DEGs enriched in “Flavonoid biosynthesis”, “Plant-pathogen interaction” and “MAPK signaling pathway-plant” were downregulated in XY, while were upregulated in QF in the same enriched pathways. In addition, “Photosynthesis (Ko00195)” and “Photosynthesis-antenna proteins (Ko00196)” were specially enriched in XY, indicating significant influence of cold stress on photosynthesis in the resistant genotype (Fig. 5A, Supplementary Table S2), while “Isoquinoline alkaloid biosynthesis (Ko00950)”, “alpha-Linolenic acid metabolism (Ko00592)”, “Tryptophan metabolism (Ko00380)” were exclusively present in QF (Fig. 5B, Supplementary Table S2).

At 24 HAT, “MAPK signaling pathway (Ko04016)” was the most significantly enriched pathway in XY, in which most DEGs were upregulated, indicating its involvement in regulating the adaptive response in the resistant genotype. “Plant hormone signal transduction (Ko04075)” and “Phenylalanine, tyrosine and tryptophan biosynthesis (Ko00400)” were significantly enriched in both XY and QF. It was interesting to note that most DEGs enriched in Plant hormone signal transduction were upregulated in XY, while were downregulated in QF, showing opposite expression pattern compared with 6 HAT (Supplementary Table S2). However, “Plant-pathogen interaction (Ko04626)”, “Glycerophospholipid metabolism (Ko00564)” and “Base excision repair (Ko03410)” were specially present in XY (Supplementary Fig. S1A), while “Phenylpropanoid biosynthesis (Ko00940)”, “Vitamin B6 metabolism (Ko00750)”, “alpha-Linolenic acid metabolism (Ko00592)” and “Biosynthesis of amino acids (Ko01230)” were exclusively enriched in QF (Supplementary Fig. S1B).

### Transcription factors (TFs) involved in responses to cold stress

**Figure 6:**
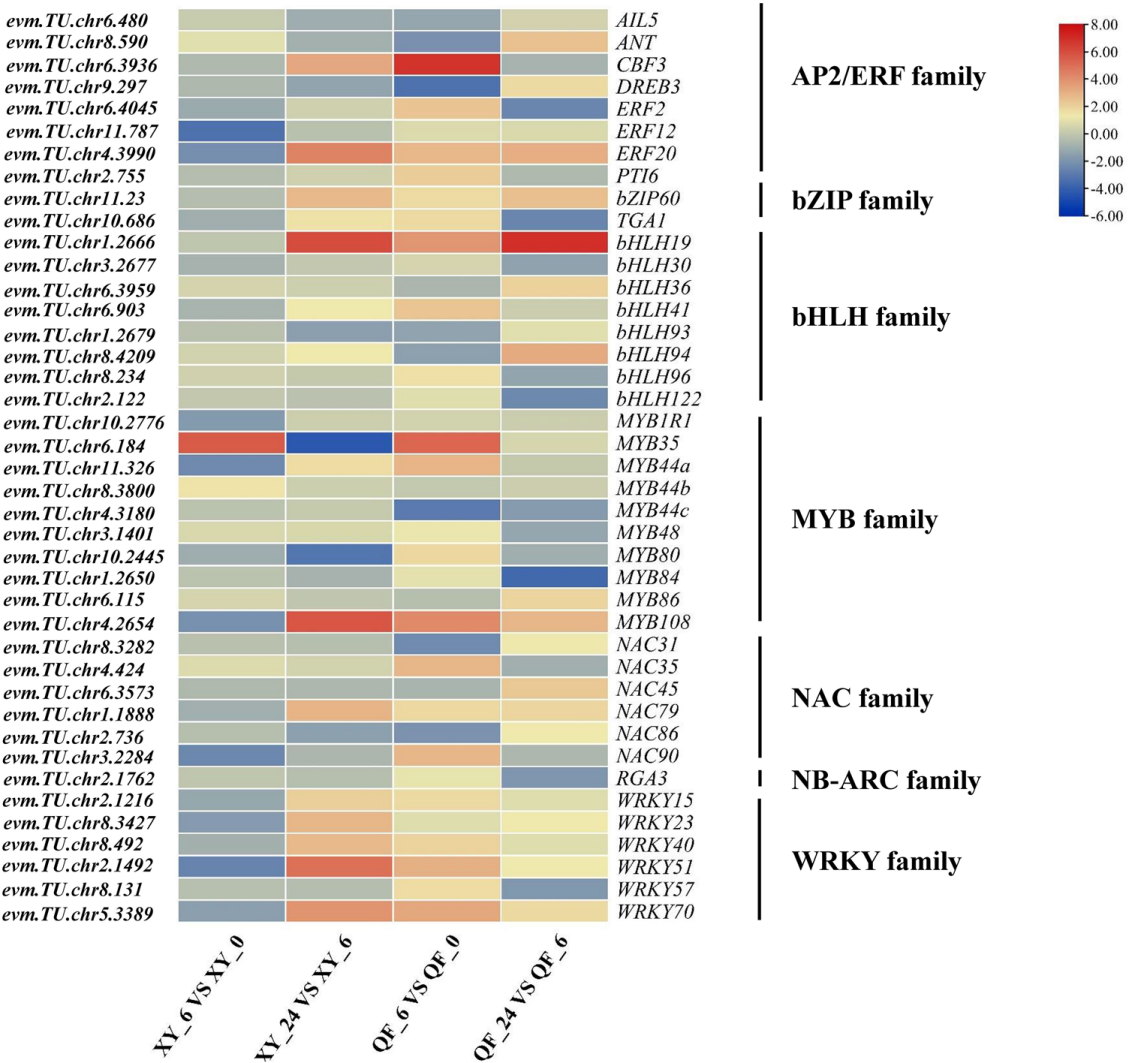
Heatmap of differentially expressed TFs under cold stress in XY and QF.

TFs play important roles in regulating the cold adaptation of plants by activating a cascade of downstream functional genes^3^. In the present study, *AP2/ERF* (62), *bHLH* (34), *bZIP* (16), *GATA* (6), *GRAS* (14), *MYB* (49), *NAM* (31), *NB-ARC* (18), *WRKY* (27) and *ZF-HD* (6) were differentially expressed between XY and QF at 6 and 24 HAT. Most transcription factors were activated by low-temperature stress except *GATA* and *ZF-HD* members (Supplementary Table S3). To infer the resistance differences between cold-tolerant and cold-sensitive genotypes, TFs showing different or opposite expression patterns between XY and QF were investigated. A total of 41 TFs were differentially expressed between genotypes, among which the *MYB* family (10 members) was the largest. Interestingly, almost all the differentially expressed *WRKY* genes were downregulated at 6 HAT but were significantly activated at 24 HAT in XY. In contrast, the expression levels of these *WRKYs* were upregulated at 6 HAT in QF (Fig. 6). Moreover, *CBF3* (*evm.TU.chr6.3936*), a key TF involved in the cold stress response, was induced in XY at 24 HAT but was activated in QF at 6 HAT.

### Cold-responsive genes related to plant hormone signal transduction

**Figure 7:**
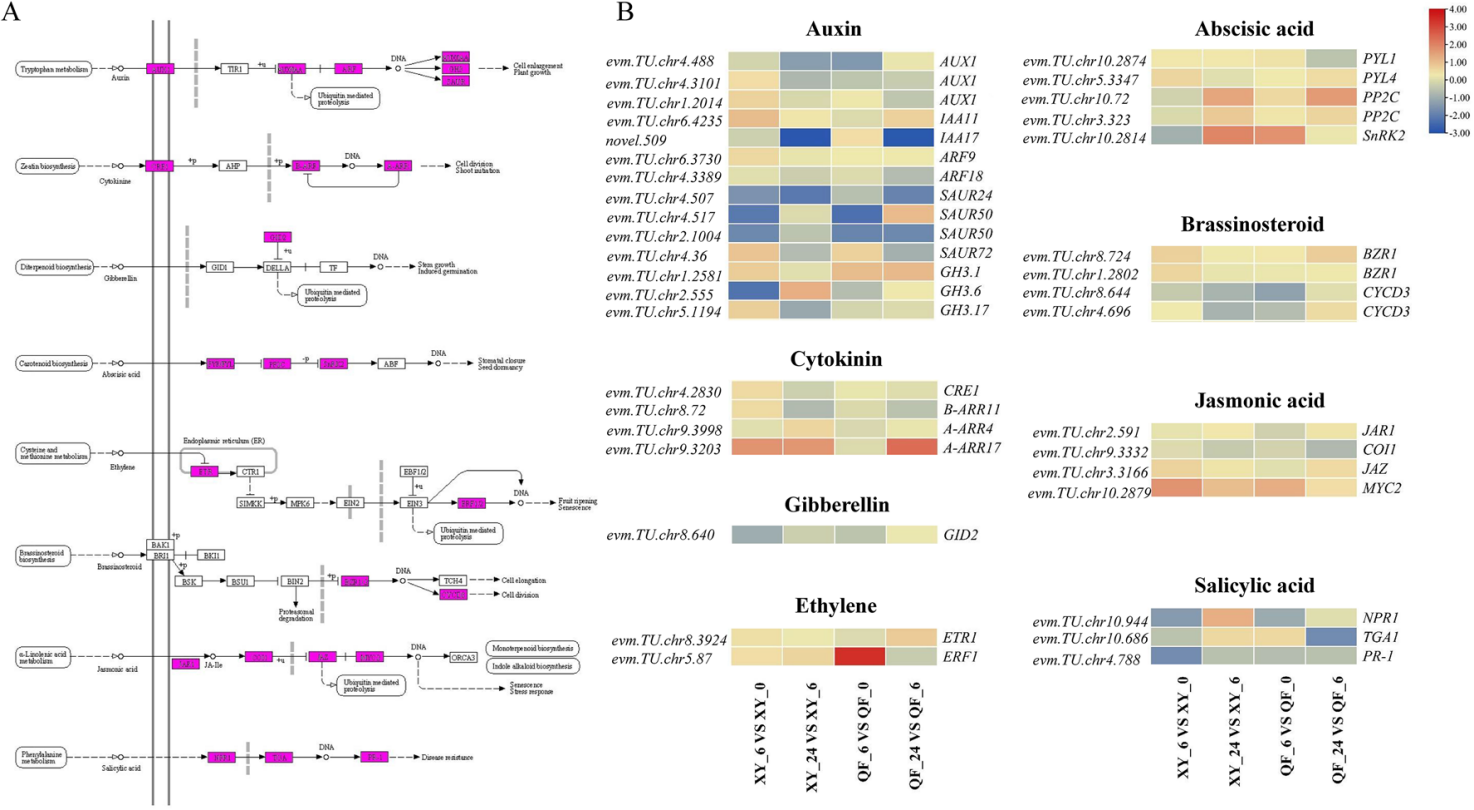
The enriched DEGs in response to cold stress in the plant hormone signal transduction pathways. (**A**) KEGG pathway analyses of the DEGs involved in eight plant hormone pathways. The pink boxes showed the enriched DEGs. (**B**) Heatmap of DEGs showing different expression patterns between XY and QF in hormone signal pathways.

Large numbers of DEGs were also enriched in plant hormone signal transduction pathways, including auxin (AUX), cytokinin (CTK), gibberellin (GA), abscisic acid (ABA), ethylene (ET), brassinosteroid (BR), jasmonic acid (JA), and salicylic acid (SA) (Fig. 7A). To elucidate the molecular basis underlying the cold tolerance difference, DEGs showing different or opposite expression patterns between QF and XY were selected for hormone signal transduction pathway analysis. Among these pathways, most DEGs were enriched in auxin compared with other pathways, including *AUX1*, *IAA*, *ARF*, *SAUR* and *GH3*. In the JA signal transduction pathway, *MYC2* (*evm.TU.chr10.2879*) and jasmonatezim-domain protein gene (*JAZ*, *evm.TU.chr3.3166*) were significantly induced in XY, and jasmonic acid-amido synthetase gene (*JAR*, *evm.TU.chr2.591*) was suppressed in QF at 6 HAT. The analysis of SA-responsive genes revealed that non-expressor of pathogenesis-related gene 1 (*NPR1*, *evm.TU.chr10.944*), *TGA* (*evm.TU.chr10.686*) and pathogenesis related protein gene (*PR1*, *evm.TU.chr4.788*) were differentially expressed between QF and XY. *NPR1* was activated in XY at 24 HAT but was suppressed in QF at both time points. Moreover, both *TGA* and *PR1* were also downregulated in QF (Fig. 7B).

Furthermore, five ABA-responsive genes showed different expression patterns between the contrasting genotypes, including pyrabactin resistance 1/pyr1-like (*PYR/PYL, evm.TU.chr10.2874* and *evm.TU.chr5.3347*), protein phosphatase 2C gene (*PP2C*, *evm.TU.chr10.72* and *evm.TU.chr3.323*) and SNF1-related protein kinases 2 gene (*SnRK2*, *evm.TU.chr10.2814*). Transcripts of most of these genes were induced in both genotypes but were activated at different time points between QF and XY. For instance, *SnRK2* expression was significantly upregulated in XY at 24 HAT; however, it was activated in QF at 6 HAT (Fig. 7B).

### Co-expression network analysis and identification of hub genes

**Figure 8:**
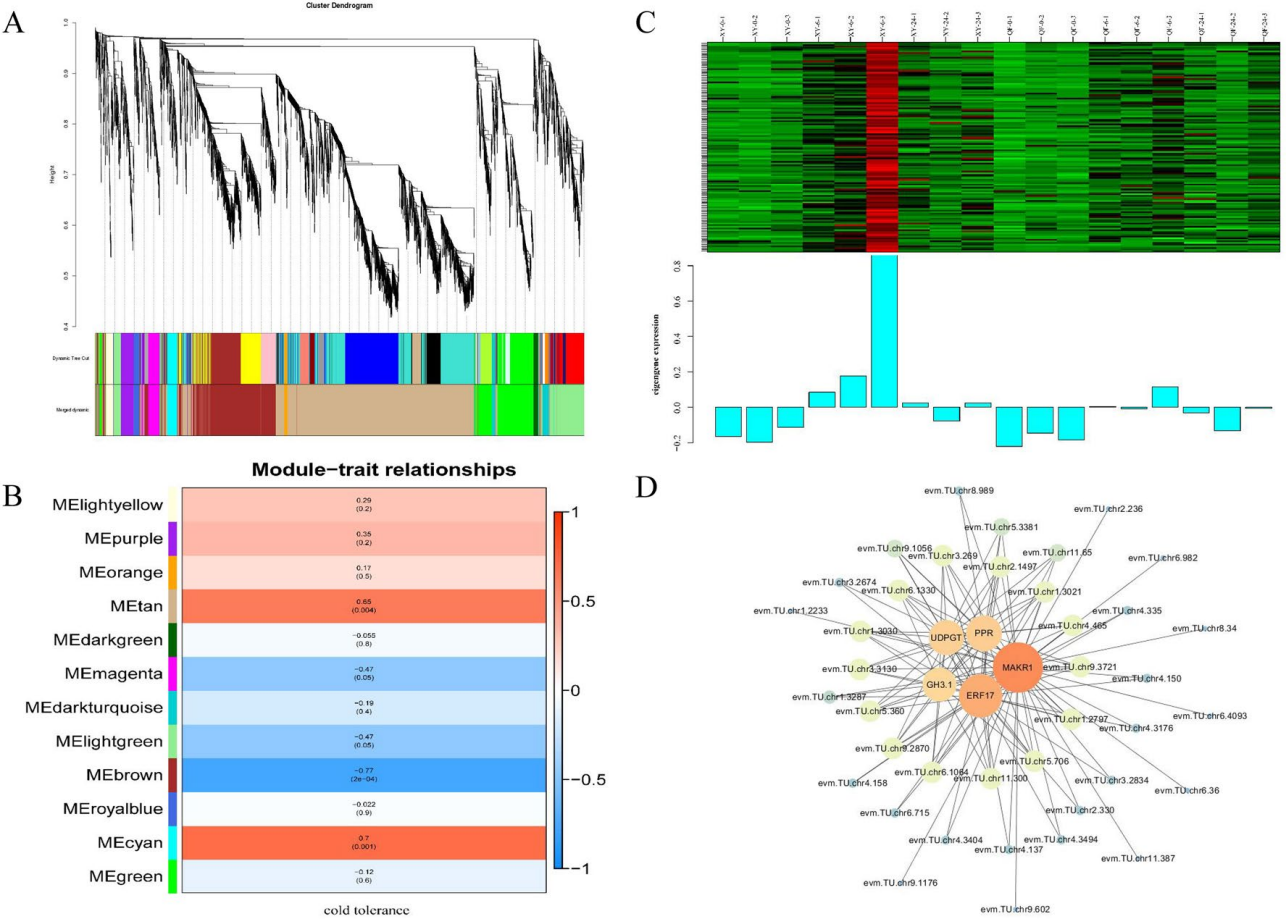
The weighted gene co-expression network analysis of DEGs in response to cold stress. (**A**) Cluster dendrogram showing co-expression modules identified by WGCNA. Each leaf in the tree represents one gene. (**B**) Module-trait relationships. The number in the box represents the correlation coefficient between modules and cold tolerance, and the number in parentheses mean the *P* value. (**C**) Gene co-expression heatmap of the cyan module (upper panel) and expression level of the corresponding eigengene in each sample (lower panel). (**D**) The co-expression network of hub genes in the cyan module visualized by Cytoscape software.

To identify gene co-expression modules and hub genes related to cold tolerance, 6,132 DEGs with FPKM > 1 were selected for co-expression network analysis using the WGCNA R package. Finally, 12 merged co-expression modules, including brown (1,202 DEGs), cyan (129 DEGs), darkgreen (61 DEGs), darkturquoise (144 DEGs), green (701 DEGs), lightgreen (537 DEGs), lightyellow (90 DEGs), magenta (171 DEGs), orange (49 DEGs), purple (169 DEGs), royalblue (68 DEGs) and tan (2,811 DEGs), were identified (Fig. 8A). Subsequently, the correlation coefficients of module-trait relationships were calculated, cyan (*r* = 0.7, *p* = 0.001) and tan (*r* = 0.65, *p* = 0.004) modules showed significant correlations with cold resistance (Fig. 8B,C).

Hub genes are highly interconnected genes in a co-expression module and are usually located upstream of a regulatory network^28^. In the present study, a total of 67 annotated hub genes (MM > 0.7, GS > 0.2) were identified in the cyan module (Supplementary Table S4). Among them, genes encoding ethylene-responsive transcription factor 17 (ERF17, evm.TU.chr11.460), pentatricopeptide repeat-containing protein (PPR, evm.TU.chr4.3274), UDP-glycosyltransferase (UGT, evm.TU.chr4.1123), indole-3-acetic acid-amido synthetase (GH3.1, evm.TU.chr1.2581) and membrane-associated kinase regulator 1 (MAKR1, evm.TU.chr7.2581) were the top five hub genes in the cyan module. Furthermore, these five hub genes and their associated genes were selected to construct a co-expression network (Fig. 8D). In this network, genes encoding pentatricopeptide repeat-containing proteins or belonging to the PPR family were the most abundant. It was interesting to note that some hub genes belonged to TFs, such as ethylene response factor 72 (*ERF72*, *evm.TU.chr5.706*). Moreover, some hub genes encoding important functional proteins were also enriched, such as auxin-responsive protein SAUR72 (*evm.TU.chr1.2233*), thaumatin-like protein (*evm.TU.chr6.4093*), E3 ubiquitin-protein ligase (*evm.TU.chr6.1064*), BON1-associated protein 2 (*evm.TU.chr5.360*) and MLP-like protein 43 (*evm.TU.chr9.2870*).

### qRT‑PCR validation of RNA‑seq results

**Figure 9:**
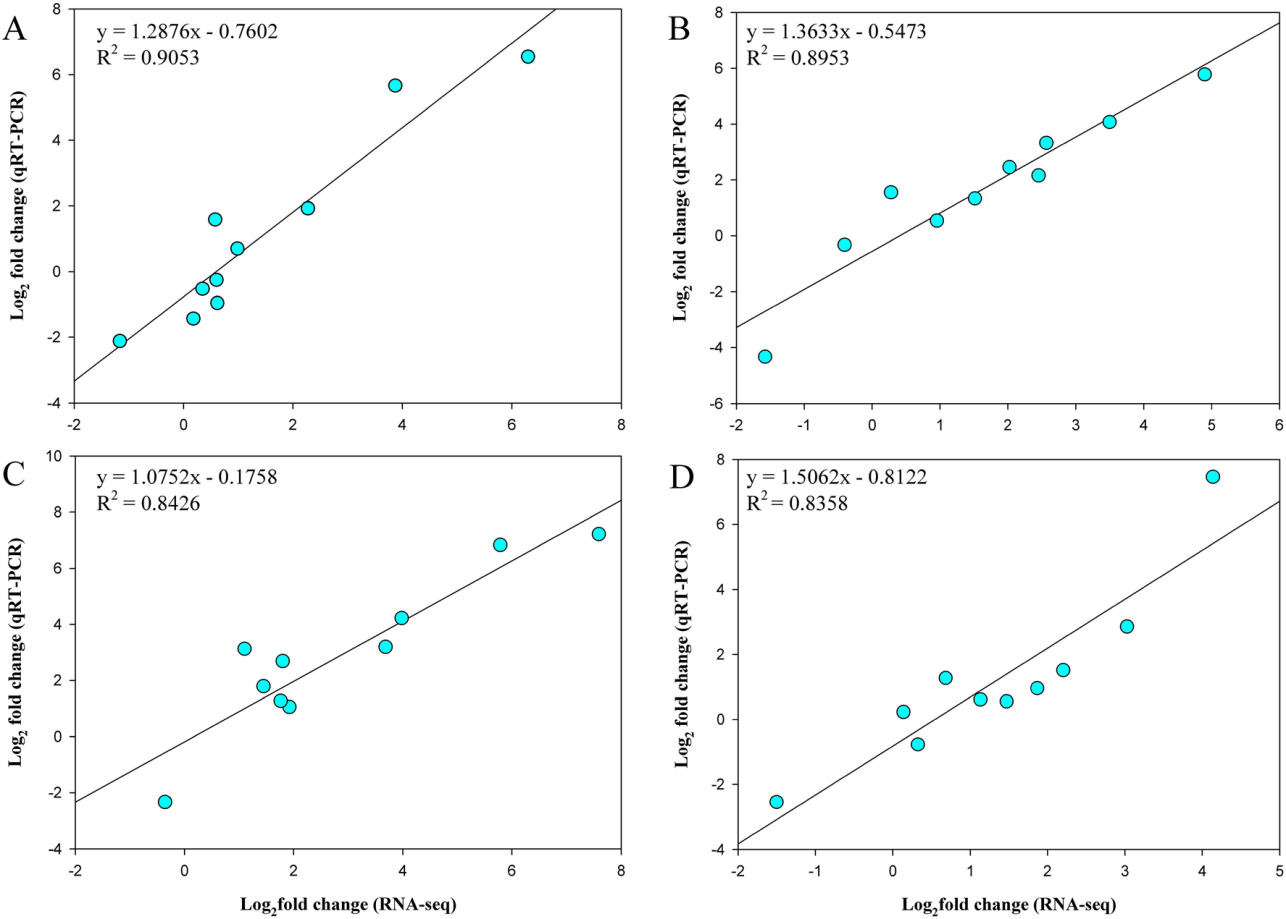
Comparisons of gene expression values obtained from RNA-seq and quantitative Real-time PCR (qRT-PCR). (**A**) XY_6 VS XY_0. (**B**) XY_24 VS XY_6. (**C**) QF_6 VS QF_0. (**D**) QF_24 VS QF_6. The R^2^ values show the correlation ratio between RNA-seq and qRT-PCR in each genotype and time point. *P* value < 0.05.

To further validate the accuracy and reliability of the RNA-seq results, 10 DEGs responsive to cold stress were randomly selected for quantitative real-time PCR (qRT-PCR). To directly compare RNA-seq data and qRT-PCR data, the relative expression levels of genes obtained from qRT-PCR were converted to log2-fold changes^29^. The results showed that the expression trends of DEGs determined by qRT-PCR were consistent with the results of RNA-seq (Fig. 9), and linear regression analysis suggested a strong correlation between RNA-seq and qRT-PCR data (R^2^ > 0.8). Therefore, it could be concluded that the results of RNA-seq were reliable.

## Discussion

Low temperature represents one of the major abiotic stresses and can severely impair plant growth, development, productivity and quality. Conversely, plants have evolved sophisticated regulatory mechanisms at the physiological and genetic levels to adapt to environmental changes. Bitter gourd, originating from tropical Asia, easily suffers from cold stress with its cultivation expanding toward high-latitude areas in China. However, very little report revealed the molecular mechanism underlying the response to low temperature and the genes regulating the cold resistance of bitter gourd. Therefore, in the present study, a comparative transcriptome analysis was performed between two bitter gourd lines with contrasting cold resistance at different time points after low-temperature treatment. In summary, a total of 7,351 DEGs were identified, involving TFs and genes participating in plant hormone signal transduction, MAPK signal transduction and other pathways.

### TFs play important roles in regulating cold resistance of bitter gourd

TFs regulate adaptive responses to low temperature by receiving upstream signals of cold stress and binding and activating cold-responsive genes downstream^30^. Consistent with findings in other plants exposed to cold stress, several TFs were also identified in the present study, including AP2/ERF, bHLH, bZIP, GATA, GRAS, MYB, NAM, NB-ARC, WRKY and ZF-HD^31^*. CBF3*, belonging to *AP2/ERF* gene family, is considered a hub in cold acclimation by regulating the expression of cold-responsive (*COR*) genes^1^. Cucumber *CBF* genes could be significantly induced when exposed to cold stress, and transgenic cucumber plants overexpressing *CsCBFs* showed stronger cold tolerance than the wild type^32^. In the present study, a *CBF3* (*evm.TU.chr6.3936*) gene was found to be upregulated under could stress in both genotypes. However, it was significantly activated at 24 HAT in XY, but was upregulated at 6 HAT in QF (Fig. 6). The different expression patterns of *CBF3* between XY and QF indicated different response mechanisms against low-temperature stress in genotypes with contrasting cold tolerance.

Previous research suggested that several *WRKY* family members could be induced by low temperature and that overexpression of *WRKYs* could enhance the cold tolerance of transgenic plants, such as *OsWRKY71* in rice^33^, *CsWRKY46* in cucumber^34^ and *PmWRKY57* in *Prunus mume*^35^. In the present study, *WRKY15* (*evm.TU.chr2.1216*), *WRKY23* (*evm.TU.chr8.3427*), *WRKY40* (*evm.TU.chr8.492*), *WRKY51* (*evm.TU.chr2.1492*) and *WRKY70* (*evm.TU.chr5.3389*) were found to be upregulated in XY at 24 HAT (Fig. 6). The expression level of *WRKY51* increased nearly 32 times at 24 HAT compared to 6 HAT in XY, but was upregulated at 6 HAT in QF. *WRKY70* was also significantly activated in the resistant genotype XY, in accordance with a report in apple, in which *MsWRKY70* was also strongly activated during the cold stress period^21^. Therefore, these two *WRKYs* in bitter gourd deserve further study to clarify their functions and roles in regulating adaptive responses to cold stress. In addition, *MYB108* (*evm.TU.chr3.3215*) also attracted our attention for its high expression in the resistant genotype at 24 HAT. Similarly, in apple and rose, *MYB108* could be induced by cold stress and overexpression of *MYB108* could enhance the cold tolerance of the transgenic plants by directly binding to the *CBF3* promoter^36,37^.

### Plant hormone involved in the adaptive response to cold stress in bitter gourd

Phytohormones are small endogenous signaling molecules and have been reported to function as vital factors to regulate plant cold resistance^38^. Our results also proposed that genes involved in plant hormone signal transduction were significantly differentially expressed under cold stress (Fig. 7). ABA has been proven to play a crucial role in enhancing plant cold tolerance, low temperature could induce the accumulation of endogenous ABA and exogenous application of ABA could improve the cold tolerance of plants^39,40^. Similarly, our results also observed a significant increase in endogenous ABA in the resistant genotype XY at 24 HAT compared with 0 HAT (Fig. 2). In ABA-dependent pathways, PYR/PYL receptor proteins can disrupt the interaction between SnRK2s and PP2Cs in the presence of ABA, thus maintaining the autophosphorylated state of SnRK2s to activate downstream transcription factors, which could induce the expression of downstream ABA-responsive genes^41^. Here, the expression levels of *PYL4*, *PP2C* and *SnRK2* were found to be increased under low temperature, in accordance with the results in tobacco^42^. Therefore, it could be concluded that the ABA-dependent signaling pathway participated in the responses to low-temperature stress in bitter gourd.

Our results also revealed that endogenous JA content in XY was significantly increased after cold treatment, while the JA level in QF was decreased after cold treatment. Previous studies have suggested that JAs are positive regulators of cold tolerance, and endogenous JA levels could be induced by cold stress in Arabidopsis and rice^43,44^. In the present study, genes associated with JA signal transduction, including *JAZ* and *MYC2,* were significantly induced under cold stress in XY at 6 HAT. MYC2, a bHLH TF, can interact with ICE1 and activate the transcription of *CBF* genes^45^. Overexpression of *MdMYC2* increased the expression levels of *CBF* genes, thus leading to enhanced cold tolerance of apple^46^. Moreover, an increasing number of studies have suggested a strong relationship between accumulated SA levels and cold tolerance in many plant species^47,48^. However, the detailed regulatory mechanism of SA in response to cold stress remains unclear. In the present study, significantly increased SA levels and differentially expressed genes involved in SA signal transduction were observed in XY under cold stress. Therefore, our results indicated that SA may participate in the regulation of cold adaptive responses of bitter gourd, and the specific mechanism needs to be further studied.

### Other important genes associated with cold resistance in bitter gourd

By WGCNA, we also identified several hub genes that were closely related to cold resistance, including *MARK1*, *ERF17*, *UGT74E2*, *GH3.1* and *PPR,* these five genes were all significantly activated in the resistant genotype at 6 HAT (Fig. 8C). *MEMBRANE-ASSOCIATED KINASE REGULATORS* (*MAKRs*) have been verified to be involved in the regulation of plant development; for example, *MAKR2* regulates root gravitropic bending and TMK-dependent rapid auxin signaling, and *MAKR4* acts in the auxin-mediated lateral root initiation process^49^. However, the specific role of *MAKR1* in regulating cold tolerance remains unreported. UGTs can respond to a variety of biological and abiotic stresses by catalyzing the glycosylation of various phytohormones and other metabolites^50^. In tea plants, *UGT78A15* and *UGT91Q2*, glycosylating eugenol glucoside and nerolidol glucoside, respectively, were strongly induced by cold stress, and downregulation of these two genes resulted in impaired cold tolerance^51,52^. *ERFs*, belonging to the *AP2/ERF* super gene family, have been demonstrated to play important roles in plant responses to cold stress by regulating downstream target genes^53^. In Arabidopsis, overexpression of *AtERF102* to *AtERF105* conferred enhanced cold tolerance to transgenic plants via the *CBF* signaling pathway^54^. *PtrERF108* could strengthen the cold tolerance of citrus by regulating the raffinose synthase encoding gene (*PtrRafS*), further increasing the content of raffinose^55^. GH3 could maintain IAA homeostasis by inactivating free IAA via conjugation of IAA with amino acids. Recently, studies have indicated that GH3 may participate in plant responses to low temperature. Ectopic overexpression of the *Stylosanthes guianensis GH3.1* gene in *Arabidopsis thaliana* increased the transcription level of *AtCBF1-3* genes and enhanced cold tolerance of transgenic plants^56^. In addition, some PPR proteins, characterized by tandem arrays of a degenerate 35-amino acid repeat motif, have also been suggested to respond to various environmental stresses, including *SVR7*, *PPR40*, *ABO5*, *PGN*, *AHG11*, *SLG1*, and *SLO2*^57^. *SOAR1*, a cytosol-nucleus dual-localized PPR, functions as a positive regulator and its overexpression lines show strong tolerance to cold stress^58^. Therefore, these hub genes could be regarded as potential cold tolerance-related genes to perform further functional characteristics and molecular mechanisms involved in regulating adaptive responses to low temperature.

## Conclusions

The present study provides comparative physiological and transcriptomic analyses between cold-resistant and cold-sensitive bitter gourd lines under low-temperature stress. After cold treatment, the significantly elevated levels of endogenous ABA, JA and SA in XY compared to those in QF indicated the involvement of the three phytohormones in regulating resistance responses to cold stress. Transcriptome analysis identified some important DEGs involved in transcription factors and plant hormone signal transduction, such as *CBF3*, *bHLHs*, *bZIPs*, *WRKYs*, *MYBs*, *PP2C*, *SnRK2* and *MYC2*. The different expression patterns between XY and QF may partly explain the cold resistance difference at the transcriptional level. Moreover, WGCNA provided a co-expression network of cold-responsive genes and identified several hub genes showing a strong relationship with cold tolerance, including *MARK1*, *ERF17*, *UGT74E2*, *GH3.1* and *PPR*. In conclusion, these findings deepen our understanding of the molecular mechanism of cold tolerance in bitter gourd and facilitate the improvement of cold-tolerant cultivars by genetic engineering methods based on potential resistance genes.

## Materials and methods

### Plant materials and cold treatment

The cold-resistant bitter gourd inbred line XiangYan (XY) and cold-sensitive line QingFeng (QF) were used as plant materials in the present study. All seeds were immersed in hot water (50 °C) for 3 h prior to sowing. Each seed was planted into a plastic pot containing sterile nursery medium (Pindstrup, Denmark). Seedlings were watered with half-strength Hoagland nutrient solution every three days and cultured in a growth chamber maintained at 28 °C (day, 16 h) and 20 °C (night, 8 h). Seedlings were exposed to 4 °C for cold treatment when they grew to the four real-leaf stage. Leaves were sampled from both lines at 0, 6 and 24 h after treatment (HAT), immediately immersed in liquid nitrogen, and then stored at -80 °C before hormone concentration determination and RNA extraction. At each time point, three biological replicates were set for each line, and five seedlings were collected for each replicate.

### Measurements of ABA, JA and SA

The extraction and quantification of endogenous ABA, JA and SA were performed according to the method with little modification^59^. In brief, 50 mg frozen leaves were ground in liquid nitrogen, and then 90% (v/v) methanol was added and homogenized by using TissueLyser. The extracts were then centrifuged at 20,000×*g* for 15 min at 4 °C, and the supernatants were collected in a new tube and evaporated in a vacuum concentrator. Residues were dissolved in 1 mL 20% (v/v) methanol and filtered through 0.22 mm nylon filter (Millipore, Bedford, MA, USA). Then the generated solutions were subjected to high performance liquid chromatography system (Agilent 1290, Santa Clara, USA) and tandem mass spectrometry (Applied Biosystems 6500 Quadrupole Trap) to detect phytohormones contents. The analytic conditions were set as follows: reversed phase column (Poroshell 120 SB-C18, 2.1 × 150 mm, 2.7 µm); 30 °C; mobile phase consisting 0.01% formic acid in H_2_O (solvent A) and 0.01% formic acid in acetonitrile (solvent B) with a flow rate of 0.8 mL/min; ionspray voltage: 4.5 kV; source temperature: 400 °C; and curtain gas: 15 Psi. To qualify ABA, JA and SA, calibration curves were constructed using internal standards of abscisic acid-d6 (ABA-d6), dehydrojasmonic acid (dhJA) and salicylic acid-d5 (SA-d5) (Olchemin Ltd, Olomouc). Each sample was qualified with three biological replicates.

### RNA extraction and sequencing

Total RNA was extracted using RNA Plant Plus Reagent (Tiangen, China) following the manufacturer’s instructions. The purity and integrity of the RNA were assessed using the NanoPhotometer® spectrophotometer (IMPLEN, CA, USA) and the RNA Nano 6000 Assay Kit of the Bioanalyzer 2100 system (Agilent Technologies, CA, USA), respectively. RNA samples with RINs ≥ 7.0 and A260/280 ratios ranging from 1.8 to 2.1 were used for library preparation.

cDNA libraries were constructed using the NEBNext® Ultra™ RNA Library Prep Kit for Illumina® (NEB, USA) following the manufacturer’s recommendations. A total of 18 libraries were generated and sequenced on the Illumina NovaSeq platform with 150 bp paired-end reads by Novogene Co., Ltd (Beijing, China).

### RNA-seq data analysis

Clean reads were obtained from the raw reads by removing reads containing adapters, unknown bases (> 10% N bases) and low-quality reads. The generated high-quality reads were mapped to the bitter gourd reference genome^60^ using HISAT2 v2.1.0 software^61^. Reads were counted using featureCounts v1.5.0, and then reads per kilo base per million mapped reads (RPKM) were calculated. Differentially expressed genes (DEGs) between two libraries were identified using DESeq2^62^. Genes with adjusted *P* value less than 0.05 and the absolute value of log2 (fold change) greater than 1 were considered significant DEGs.

To characterize the functional annotation of DEGs, a BLASTX alignment was performed by comparing with the NCBI non-redundant protein database (Nr), Clusters of Orthologous Groups of proteins database (COG; http://www.ncbi.nlm.nih.gov/COG/), Pfam database (http://pfam.sanger.ac.uk), Kyoto Encyclopedia of Genes and Genomes (KEGG; http://www.genome.jp/kegg/) and Swiss-Prot database(http://www.ebi.ac.uk/uniprot/) .Gene Ontology (GO; http://www.geneontology.org/) annotation was also implemented using the clusterProfiler R package and GO terms with *P* values less than 0.05 were considered significantly enriched.

### Co-expression network analysis and hub gene identification

To further investigate the regulatory mechanism and hub genes in response to cold stress, weighted gene co-expression network analysis (WGCNA) was performed based on the gene expression levels of 18 samples using the WGCNA R package^63^. The R package DCGL was employed to filter genes, and genes with FPKM values larger than 1 were selected for co-expression analysis. A value of 5 was determined as the appropriate threshold parameter β to satisfy the scale-free network distribution principle. Then, the adjacency matrix was used to calculate the topological overlap matrix (TOM), and the gene connection network was constructed. Gene modules were clustered and identified through dynamic tree cut algorithm with the following settings: TOMType, unsigned; mergeCutHeight, 0.25; minModuleSize, 30. The correlation coefficients between module eigengenes and cold resistance were calculated, and module–trait value *r* ≥ 0.5 was determined as a vital module. The module eigengene (MM) and gene significance (GS) values were used to reflect the correlation between the gene expression and the corresponding module eigengene or the phenotype data, respectively. In the present study, genes with MM ≥ 0.7 and GS ≥ 0.2 were considered hub genes in the vital module. The co-expression gene network results were visualized by Cytoscape software (v3.7.2).

### Quantitative real-time PCR validation

To confirm the RNA-seq results, quantitative real-time PCR (qRT-PCR) was performed on a selection of 10 genes in response to cold stress revealed by RNA-seq. All qPCR experiments were repeated with three biological replicates. RNA was extracted from leaves of both XY and QF using the RNAprep Pure Plant Kit (Tiangen, China). cDNA was synthesized using HiScript II QRT Super Mix (Vazyme, Nanjing, China) following the manufacturer’s protocol. Gene-specific primers were designed by the online tool Primer 3.0 and detailed information was listed in Supplementary Table S5. The 20 µL PCR reaction system contained 1 µL of cDNA, 0.5 µL each of forward and reverse primers (10 µM), 10 µL of 2 × ChamQ Universal SYBR qPCR Master mix (Vazyme) and 8 µL of ddH_2_O. qPCR was performed on the Roche LightCycler480 under the following conditions: 95 °C for 30 s, followed by 40 cycles of 95 °C for 10 s, 60 °C for 10 s, and 72 °C for 20 s. A melting curve was generated to determine the amplification specificity and reaction contamination. The relative expression levels of the target genes were calculated using the 2^−ΔΔCt^ method^64^. To directly compare data between RNA-seq and qPCR, relative expression values were converted to log_2_ fold change of transcript abundance.

### Ethics statement

All experiments conducted with plants were carried out according to relevant institutional, national, and international guidelines and legislation.

### Sample collection

The cold-resistant bitter gourd XiangYan (XY) and cold-sensitive bitter gourd QingFeng (QF) are inbred lines that were preserved and provided by Institute of Vegetable Crops (IVC), Jiangsu Academy of Agricultural Sciences (JAAS). None of these two lines was wild plant nor endangered species. The authors are permitted to conduct scientific research using these two bitter gourd lines.

### Supplementary Information

Supplementary Figure S1.

Supplementary Table S1.

Supplementary Table S2.

Supplementary Table S3.

Supplementary Table S4.

Supplementary Table S5.

## Data Availability

The sequencing data was deposited in the National Center for Biotechnology Information (NCBI) and can be accessed via BioProject ID PRJNA943061.
